# Primary mediastinal ectopic thyroid in a 43-year-old woman: A diagnostic and surgical challenge – a case report and literature review

**DOI:** 10.1097/MD.0000000000050579

**Published:** 2026-09-04

**Authors:** Zheng Wang, Shuo Liang, Kunpeng Li, Xike Lu, Xin Li, Daqiang Sun

**Affiliations:** a Department of Thoracic Surgery, Tianjin Chest Hospital (Affiliated Hospital of Tianjin University), Tianjin, China; b Department of Radiology, Tianjin Chest Hospital (Affiliated Hospital of Tianjin University), Tianjin, China; c Department of Pathology, Tianjin Chest Hospital (Affiliated Hospital of Tianjin University), Tianjin, China.

**Keywords:** case report, ectopic thyroid, median sternotomy, mediastinum

## Abstract

**Rationale:**

Primary mediastinal ectopic thyroid (PMET) is exceedingly rare, and its imaging features often overlap with those of thymic epithelial tumors, lymphoma, Castleman disease, and paraganglioma. The absence of anatomical continuity with the cervical thyroid and the presence of an independent intrathoracic blood supply are important considerations in differential diagnosis and surgical planning. This report aims to highlight the diagnostic value of thyroid-like enhancement and the absence of cervical continuity and to demonstrate how preoperative computed tomography angiography (CTA)-based vascular mapping can guide selection of the surgical approach and intraoperative vascular control in PMET.

**Patient concerns:**

An asymptomatic 43-year-old woman was admitted after routine chest computed tomography (CT) revealed an anterior mediastinal mass. Physical examination showed no palpable cervical mass or thyromegaly, and thyroid function was normal.

**Diagnoses:**

Contrast-enhanced CT demonstrated an irregular soft-tissue lesion measuring approximately 49 × 37 × 56 mm, with heterogeneous density, scattered calcifications, and non-enhancing hypodense areas. The mass showed intense but heterogeneous enhancement and lacked anatomical continuity with the cervical thyroid. CTA revealed abundant vascularity, with multiple tortuous small-caliber vessels coursing over the tumor surface adjacent to the left innominate vein. Intraoperatively, no continuity with the cervical thyroid was observed. Histopathological examination confirmed benign ectopic thyroid tissue.

**Interventions:**

Based on the vascular anatomy and tumor location, median sternotomy was performed. The tumor, together with part of the adjacent thymus, was completely resected after sequential clipping and division of the feeding vessels using titanium clips and ultrasonic shears.

**Outcomes:**

The postoperative course was uneventful. At approximately 11 months after surgery, follow-up chest CT showed no evidence of recurrence, thyroid function remained normal, and cervical ultrasonography revealed no suspicious abnormalities.

**Lessons:**

PMET should be considered in the differential diagnosis of hypervascular anterior mediastinal masses, particularly when imaging demonstrates thyroid-like enhancement without anatomical continuity with the cervical thyroid. Preoperative CTA can delineate tumor vascularity and its relationship with adjacent mediastinal vessels, thereby facilitating selection of the surgical approach and planned vascular control. Complete excision is generally associated with a favorable outcome, although continued postoperative surveillance remains appropriate.

## 1. Introduction

Ectopic thyroid tissue (ETT) is generally uncommon, with an estimated prevalence of approximately 1 per 100,000 to 300,000 in the general population, rising to 1 per 4000 to 8000 among individuals with thyroid disease; the most frequent locations are the tongue base and the midline of the neck.^[1]^ Mediastinal ETT is even rarer and is typically reported only as isolated cases or small series.^[2]^

On imaging, mediastinal ETT may show “thyroid-like” attenuation and enhancement; however, these features overlap considerably with thymic epithelial tumors, lymphoma, germ cell tumors, and neurogenic tumors, leading to frequent misclassification as common anterior mediastinal neoplasms or metastases. Radionuclide scintigraphy can support the diagnosis but is not universally available in practice.^[3]^ Crucially, mediastinal ETT must be distinguished from retrosternal thyroid extending from the neck. A truly “primary” mediastinal ETT (PMET) lacks any anatomical continuity with the cervical thyroid – cases that, in recent years, have still been reported sporadically.

Recent data from a single-center series of 7 patients underscored the heterogeneity of this entity: clinical presentation, anatomic location, and operative approach varied. PMETs were predominantly supplied by intrathoracic vessels, and preoperative identification of vascular supply (e.g., with computed tomography angiography [CTA]) informed the choice of surgical approach and perioperative risk management, necessitating individualized decisions based on imaging and perioperative assessment.^[4]^

Here, we report a left anterior mediastinal PMET in which both imaging and intraoperative findings confirmed the absence of continuity with the cervical thyroid. The lesion was adjacent to the left innominate vein, with multiple tortuous, small intrathoracic vessels traversing its surface. In conjunction with recent literature, we discuss radiologic recognition, differential diagnosis against common anterior mediastinal tumors, and a blood-supply–oriented surgical strategy.

## 2. Case presentation

**Figure 1.**
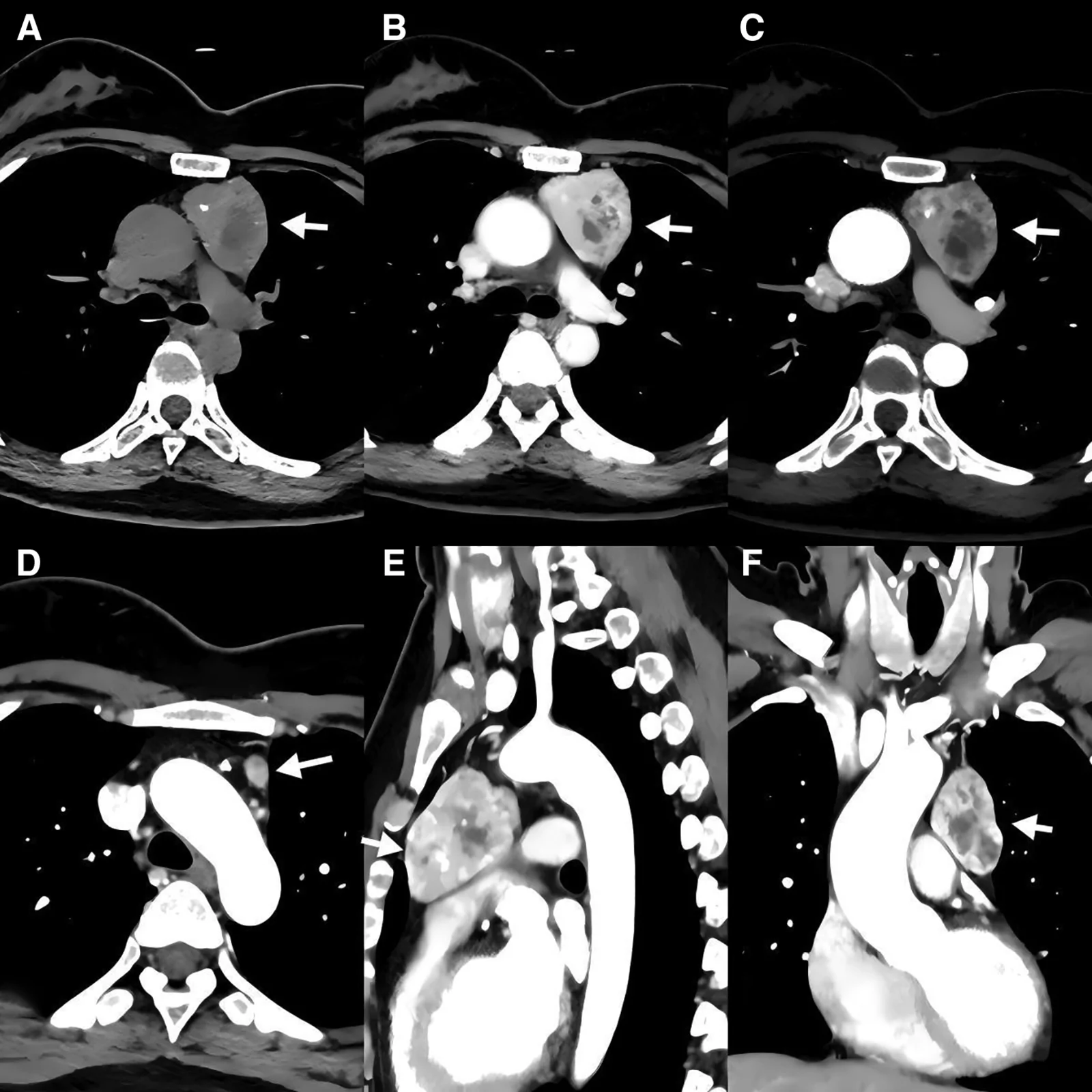
Chest CT and CTA findings of the anterosuperior mediastinal mass (white arrows). (A) Non-contrast axial CT: mean attenuation of the solid component 75.82 ± 18.46 HU. (B) Arterial-phase axial CT: marked, heterogeneous enhancement. (C) Venous-phase axial CT: partial washout. (D–F) Multiplanar reformations (axial, sagittal, coronal) and CTA demonstrate no anatomical continuity with the cervical thyroid and close adjacency to the left innominate vein; small-caliber vessels coursing along the superior aspect of the lesion are visualized on CTA (arrows). CT = computed tomography, CTA = computed tomography angiography, HU = Hounsfield unit.

An asymptomatic 43-year-old woman was admitted after an anterior mediastinal mass was detected on chest computed tomography (CT) during routine health screening. On admission, the patient was afebrile with stable vital signs (blood pressure 122/76 mm Hg, heart rate 78 beats/min, respiratory rate 18/min, SpO_2_ 99% on room air). No cervical masses or thyromegaly were palpable. Chest inspection and auscultation were unremarkable, with no abnormal breath sounds or murmurs detected. No peripheral lymphadenopathy or other systemic abnormalities were observed. No pertinent medical comorbidities, family history, or psychosocial factors were identified. On admission, contrast-enhanced chest CT revealed an irregular soft-tissue lesion in the anterior mediastinum measuring approximately 49 × 37 × 56 mm, with heterogeneous density, scattered calcifications, and hypodense areas. The non-contrast image showed relatively high attenuation of the solid component (Fig. 1A). The lesion demonstrated marked heterogeneous enhancement during the arterial phase (Fig. 1B), followed by partial washout during the venous phase (Fig. 1C). Multiplanar reformations demonstrated no anatomical continuity with the cervical thyroid and showed the close relationship between the lesion and the left innominate vein (Fig. 1D–F). Both chest CT and CTA were performed on a Siemens SOMATOM Definition Flash dual-source CT scanner (Siemens Healthineers, Germany). Scan parameters were: tube voltage 120 kVp; tube current 200 to 250 mAs; slice thickness 5 mm; interslice gap 5 mm; and reconstruction thickness 1 mm using a medium-sharp kernel. Contrast-enhanced images were acquired after intravenous injection of iohexol (iodine concentration 350 mg I/mL) via an antecubital vein using a power injector (dose 80 mL; flow rate 3.0 mL/s). The arterial phase was triggered at an ascending aorta threshold of 100 HU (Hounsfield unit), and the venous phase was obtained with a 60-second delay. Quantitative measurements were performed on non-contrast, arterial, and venous phase images by placing 3 non-overlapping ROIs within the solid portion of the lesion, avoiding calcifications, necrosis, and vascular artifacts; mean and standard deviation (mean ± SD) values were recorded and used to analyze the enhancement curve. The results were: non-contrast, 75.82 ± 18.46 HU; arterial phase, 177.39 ± 64.46 HU; venous phase, 103.73 ± 30.30 HU. The preliminary radiologic impression was thymoma, with no visible continuity with the cervical thyroid.

Given the lesion’s hypervascularity, paraganglioma, Castleman disease, and ectopic thyroid were included in the differential diagnosis. Preoperative thyroid function tests were within normal limits: T3 1.77 nmol/L, T4 124.00 nmol/L, FT3 4.24 pmol/L, FT4 18.20 pmol/L, TSH 2.070 mIU/L. Routine serum biochemistry was unremarkable. CTA demonstrated multiple tortuous small-caliber vessels coursing along the superior aspect and surface of the lesion (Fig. 1D–F). The vascular supply could not be traced to cervical thyroidal arteries, suggesting independent intrathoracic feeders.

**Figure 2.**
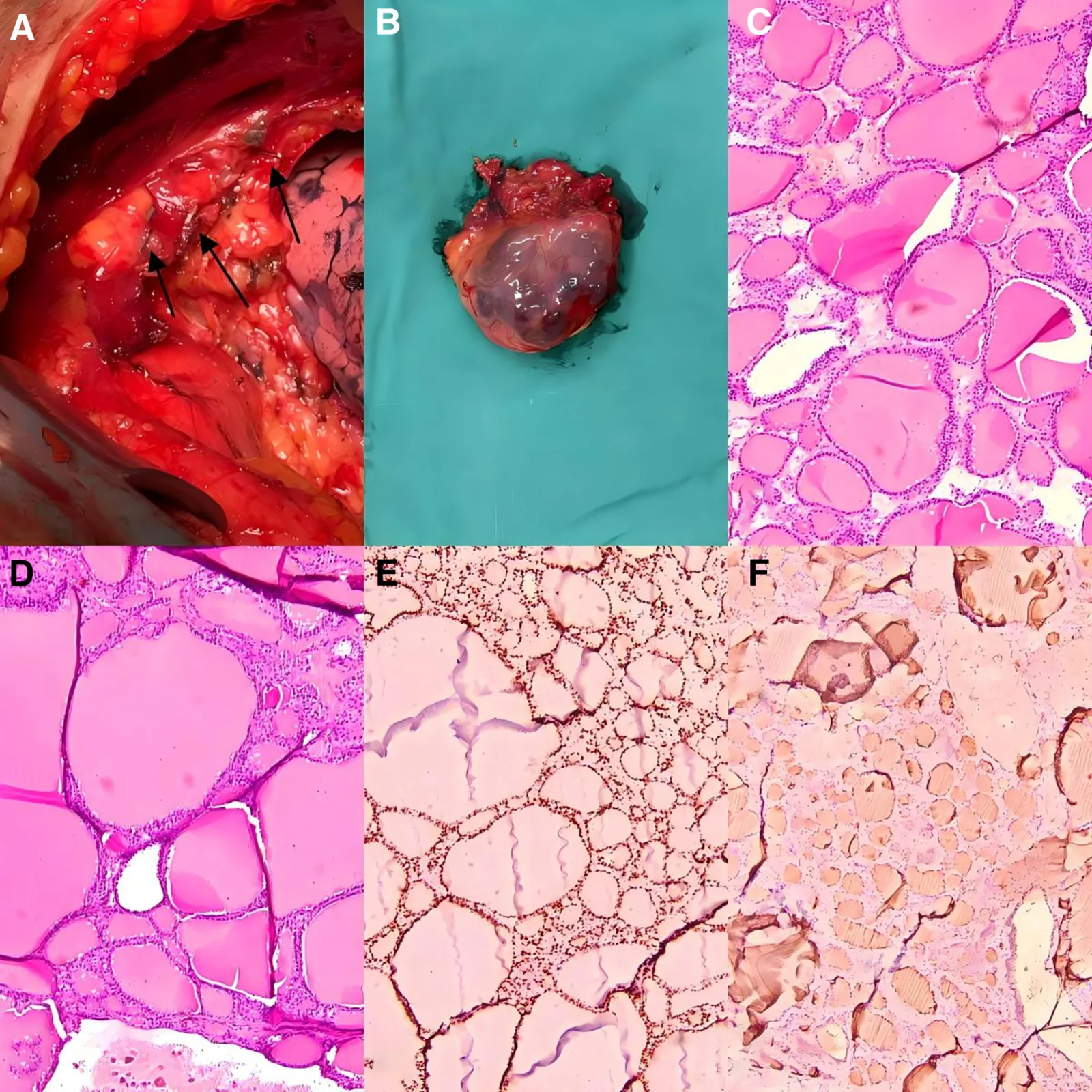
Intraoperative and pathological features. (A) Intraoperative photograph: Black arrows indicate feeding vessels secured with titanium clips. (B) Gross specimen: Cystic–solid mass with an intact capsule; resected left anterior mediastinal tissue measuring 10 × 8 × 5 cm, containing a solid nodule of 5.5 × 4.5 × 4 cm. (C) Low-power view (H&E, ×40): Variably sized follicles, some with cystic dilatation and fibrous septa; colloid of varying amounts within the lumina. (D) High-power view (H&E, ×400): Follicular epithelium ranging from flattened to cuboidal/columnar; no capsular or vascular invasion and no classic malignant nuclear features. (E) Immunohistochemistry: Diffuse nuclear positivity for TTF-1 in follicular epithelial cells. (F) Immunohistochemistry: Diffuse cytoplasmic positivity for thyroglobulin (Tg).

Based on the vascular characteristics and tumor location, a median sternotomy was selected. Under single-lumen endotracheal intubation with conventional ventilation, intraoperative inspection confirmed a hypervascular anterior mediastinal mass with multiple small surface vessels in proximity to the left innominate vein and no anatomical continuity with the cervical thyroid. Vascular control followed a proximal-control–first strategy: each feeder was proximally clipped with titanium clips and then divided using ultrasonic shears (Fig. 2A). After vascular control, the mass was completely resected en bloc together with adjacent thymic tissue. Estimated blood loss was approximately 30 mL; operative time was 74 minutes.

Gross examination showed a capsulated cystic-solid mass with an intact thin capsule (Fig. 2B). The resected left anterior mediastinal specimen measured 10 × 8 × 5 cm and contained a gray-brown, slightly firm solid nodule measuring 5.5 × 4.5 × 4 cm. Microscopically, variably sized thyroid follicles, some with cystic dilatation and fibrous septa, were observed at low power, with varying amounts of colloid within the follicular lumina (Fig. 2C). At high power, the follicular epithelium ranged from flattened to cuboidal or columnar, with no capsular or vascular invasion and no classic nuclear features of papillary thyroid carcinoma (Fig. 2D). Immunohistochemistry demonstrated diffuse nuclear positivity for TTF-1 in follicular epithelial cells (Fig. 2E) and diffuse cytoplasmic positivity for thyroglobulin (Fig. 2F). Ki-67 was <1%. The overall morphology and immunophenotype were consistent with benign ETT.

On postoperative day 6, thyroid function was: T3 1.42 nmol/L, T4 120.00 nmol/L, FT3 3.93 pmol/L, FT4 18.30 pmol/L, TSH 1.530 mIU/L (Table 1). Preoperative thyroid ultrasonography had not been performed. Prompted by the postoperative pathological diagnosis of ectopic thyroid, cervical ultrasound was obtained, showing overall normal morphology and echotexture of both lobes, with small C-TIRADS 2 to 3 nodules in the left lobe; fine-needle aspiration was not performed. Whole-body thyroid scintigraphy was not obtained pre- or postoperatively. The postoperative course was uneventful, without notable complications. The patient reported relief after being informed of the benign pathological diagnosis and returned to normal daily activities without persistent discomfort. At the most recent follow-up on July 2, 2026, approximately 11 months after surgery, she remained clinically well. Follow-up chest CT showed no evidence of local recurrence or other abnormal findings. Thyroid function tests remained within the reference ranges, and cervical ultrasonography showed no suspicious lesion or clinically significant interval change. No delayed postoperative complications were observed. The clinical course and key events are summarized in Table 2.

**Table 1:** Perioperative and follow-up thyroid function tests.

Indicator (unit)		Preoperative	Post-op Day 6	Post-op Month 11	Reference range
T3 (nmol/L)	1.77		1.42	1.87	1.3–3.1
T4 (nmol/L)	124.00		120.00	135	66–181
FT3 (pmol/L)	4.24		3.93	5.12	3.5–6.5
FT4 (pmol/L)	18.20		18.30	19.8	11.5–22.7
TSH (mIU/L)	2.07		1.53	2.32	0.27–4.2

## 3. Discussion

The vast majority of mediastinal thyroid tissue represents retrosternal extension from the lower pole of the cervical gland and therefore maintains anatomical continuity with the thyroid. In contrast, a truly primary mediastinal ectopic thyroid (PMET) lacks any continuity with the cervical thyroid and remains sparsely reported, mostly as isolated case reports.^[5]^ In our patient, both imaging and intraoperative findings confirmed the absence of continuity with the cervical gland. The diagnosis of PMET was supported by 3 principal findings: imaging and intraoperative inspection demonstrated no anatomical continuity with the orthotopic cervical thyroid; histopathology showed benign thyroid follicular tissue with colloid, supported by diffuse TTF-1 and thyroglobulin expression; and postoperative cervical ultrasonography confirmed the presence of an orthotopic thyroid gland without a suspicious primary malignant lesion. The preserved postoperative thyroid function further indicated that the mediastinal lesion was not the patient’s only functioning thyroid tissue.

Clinical manifestations of mediastinal ectopic thyroid are nonspecific; many lesions are discovered incidentally on chest CT, while some patients present with compressive symptoms such as chest tightness, dyspnea, or dysphagia, and most have normal thyroid function. Radiologically, mediastinal ETT often appears as a soft-tissue mass – sometimes with calcification or cystic change – and shows heterogeneous, sometimes “thyroid-like,” enhancement. However, these features substantially overlap with those of thymoma, Castleman disease, lymphoma, and paraganglioma, making preoperative diagnosis challenging.^[3,6]^ Radionuclide scintigraphy that demonstrates mediastinal uptake with a normal cervical gland can facilitate diagnosis, but it is not always obtainable in clinical practice.^[3]^ Consequently, preoperative definitive diagnosis is challenging and ultimately relies on pathology. Neoplastic transformation arising in ETT – benign or malignant – is uncommon.^[7]^ Nevertheless, because of the risks of malignant change, progressive enlargement, intralesional hemorrhage with potential airway compromise, and compression of vital mediastinal structures, surgical excision is recommended.

The vascular supply is another salient characteristic of PMET. Unlike retrosternal extension, which is mainly supplied by the inferior thyroid arteries, PMETs are typically vascularized by intrathoracic vessels such as the internal thoracic artery, the innominate artery, or branches of the aorta. In our case, CTA clearly depicted adjacency to the left innominate vein with multiple tortuous, small intrathoracic vessels coursing over the tumor surface; intraoperative findings were concordant. We controlled each feeder with titanium clips followed by division using ultrasonic shears, which facilitated a safe and orderly resection. These observations reiterate the importance of preoperative vascular mapping.

Surgical resection remains the mainstay of treatment for mediastinal ectopic thyroid. The choice of approach depends on tumor location, size, relationships with adjacent structures, and vascularity. Minimally invasive approaches may be appropriate for selected lesions with favorable anatomical and vascular characteristics.^[4,8]^ However, conversion to an open approach because of intraoperative bleeding has also been reported.^[4]^ In our patient, the close relationship with the left innominate vein and the presence of numerous small-caliber vessels favored median sternotomy rather than a minimally invasive approach.

This case has certain limitations. First, preoperative cervical ultrasonography was not performed, which could have aided early assessment for a cervical primary lesion. Postoperative ultrasound, prompted by the pathological diagnosis, revealed only small C-TIRADS 2 to 3 nodules without suspicious malignancy. Second, neither pre- nor postoperative ^99mTc or ^123I scintigraphy was obtained, limiting direct functional confirmation of thyroid tissue. That said, radioiodine uptake is not invariably observed in ETT, and the diagnostic utility of scintigraphy may be constrained in this context.^[3]^ Despite these gaps, the diagnostic chain remains coherent: imaging and intraoperative findings excluded continuity with the cervical thyroid; CTA demonstrated independent intrathoracic feeders; histology and immunohistochemistry (TTF-1 and Tg positivity with Ki-67 < 1%) supported thyroid follicular origin; postoperative thyroid function stayed stable, and follow-up imaging has shown no recurrence. Despite these limitations, the diagnosis was supported by the absence of anatomical continuity with the cervical thyroid on imaging and intraoperative inspection, histopathological and immunohistochemical confirmation of thyroid follicular tissue, and postoperative ultrasonographic confirmation of an orthotopic thyroid gland without a suspicious primary lesion. Approximately 11 months after surgery, chest CT showed no evidence of recurrence, thyroid function remained within the reference ranges, and cervical ultrasonography revealed no suspicious abnormalities. Nevertheless, longer-term follow-up remains necessary.

## 4. Conclusion

Although PMET is rare, it should be considered in the differential diagnosis of hypervascular anterior mediastinal masses, particularly when imaging demonstrates thyroid-like enhancement without anatomical continuity with the cervical thyroid. Preoperative CTA can delineate tumor vascularity and its relationship with adjacent mediastinal vessels, thereby facilitating selection of the surgical approach and planned vascular control. Complete surgical excision is generally associated with a favorable outcome. In the present case, no recurrence, thyroid dysfunction, or suspicious cervical thyroid abnormality was identified during approximately 11 months of follow-up; however, continued surveillance is warranted because longer-term outcome data remain limited and malignant transformation, although uncommon, has been reported.^[9]^

**Table 2:** Timeline.

Date (relative)	Event
Day −3	Routine chest CT during health check revealed an anterior mediastinal mass.
Day 0	Admission; CTA confirmed hypervascular mediastinal mass without cervical continuity.
Day 3	Median sternotomy and complete surgical resection performed.
Post-op Day 6	Thyroid function tests normal; cervical ultrasound showed only benign C-TIRADS 2–3 nodules.
Post-op Month 11	Follow-up chest CT showed no evidence of recurrence. Thyroid function remained within the reference ranges, and cervical ultrasonography revealed no suspicious abnormalities

## Author contributions

**Conceptualization:** Zheng Wang.

**Data curation:** Shuo Liang, Kunpeng Li.

**Funding acquisition:** Daqiang Sun.

**Investigation:** Zheng Wang.

**Project administration:** Xike Lu.

**Resources:** Shuo Liang.

**Supervision:** Xike Lu, Xin Li, Daqiang Sun.

**Writing – original draft:** Zheng Wang.

**Writing – review & editing:** Daqiang Sun.
